# The Journal—Close of Volume X

**Published:** 1895-06

**Authors:** 


### EDITORIAL DEPARTMENT.

F. E. DANIEL, M. D., Editor.
S. E. HUDSON, M. D., Managing Editor.
A. J. SMITH. M. D., Galveston, Associate Editor.

EDITORIAL STAFF:

PROF. J. E. THOMPSON, M. D., Texas Medical College, Galveston; Surgery.
PROF. WM. KEILLER, M. D., Texas Medical College, Galveston; Obstetrics and Gynecology.
PROF. DAVID CERNA, M. D., Texas Medical College, Galveston; Therapeutics.
PROF. A. J. SMITH, M. D., Texas Medical College, Galveston; Medicine
DR. R. H. L. BIBB, Saltillo, Mexico; Foreign Correspondent.

Official organ of the West Texas Medical Association, the Houston District Medical Association, the Austin District Medical Society, the Galveston County Medical Society, and several others

### THE JOURNAL—CLOSE OF VOLUME X.

In rounding up at the close of the tenth year, and taking stock in a general way, the JOURNAL has much to congratulate itself on, and little to regret. That the JOURNAL has prospered, is evident; it has fattened up, and looks sleek; that's the way with individuals. Its business end has been held up, and it pays (incidentally that's what most journals are conducted for). In the main, its object has been accomplished. It has done much towards the organization of the profession, and in inciting to a higher plane of professional life; the line has been sharply drawn between the regular and the irregular, and the quack has been made to know his place. There is yet much to be done, and encouraged by the generous support, moral and financial, that the JOURNAL has received from the better element of the profession, it addresses itself anew to the task. We must have a better medical law and a higher standard of medical education, and better organization of the State Association.

In one sense the JOURNAL is a departure from the beaten track. It is not merely a medical journal, filled with dry details of medicine; but it is a PHYSICIANS' MAGAZINE; it is to the doctor what the daily paper is to the business man. It gives the news of interest to doctors; and discusses such topics as doctors like to talk about. Its contents are varied; and really, a fair estimate of its character and value can not well be made from reading one number. In the course of a year there is great variety. Many subjects germane to medicine, but still, not the dry details of practice, have been discussed, embracing such topics as physicians most like to discuss in their leisure hours.

We call on old friends of the energetic and go ahead little "Red-back," to make its merits known to those of their doctor friends who are not subscribers; to canvas for us; spread its influence—help it to throw its benign light into new places. It is doing a missionary work; it is doing good,—shove it along!

We can not close this brief blast of our bazoo without an acknowledgement and a bow to all those who have assisted us. We know our friends and love them, and we are always glad to hear from them (especially when there is a postal order in the letter).

The Texas star is in the ascendant; the decks have been cleared for action, and we will now go in to win, on the ELEVENTH ROUND.

No journal in the United States is more quoted from, or oftener referred to by its contemporaries than the Texas Red-back; no journal exerts a better or a wider influence; no doctor should be without it. The traveling men—drummers for drug houses—tell us they see it everywhere they go, and it is like meeting an old acquaintance. Now is the time to subscribe; you can pay in the fall—when you sell your potatoes.

